# The Chemical Stability Characterization and Kinetics of Statins in Aqueous Cyclodextrin Ocular Preparations: A Formulation Perspective

**DOI:** 10.3390/pharmaceutics17070808

**Published:** 2025-06-23

**Authors:** Ismael Abo Horan, Thorsteinn Loftsson, Hakon Hrafn Sigurdsson

**Affiliations:** Faculty of Pharmaceutical Sciences, University of Iceland, Hofsvallagata 53, 107 Reykjavik, Iceland; iah8@hi.is (I.A.H.); thorstlo@hi.is (T.L.)

**Keywords:** statins, cyclodextrins, rate constants, ocular drug delivery

## Abstract

**Background**: Topical statin therapy holds promise for ocular diseases, such as age-related macular degeneration, but the effective delivery to the posterior segment is limited by poor aqueous solubility, chemical instability, and ocular barriers. Cyclodextrins (CDs) can enhance statin solubility and stability; however, the behavior of CD–statin complexes in aqueous eye drops—particularly their influence on the equilibrium between the inactive lactone (ring closed) and active hydroxyacid forms (ring open)—remains unclear. This study aimed to (i) investigate how 5% and 10% (*w*/*v*) concentrations of selected CDs affect the lactone/acid equilibrium of simvastatin and atorvastatin and (ii) define formulation parameters (statin form, CD type and concentration, and pH range) for stable eye drop development. **Methods**: Simvastatin or atorvastatin was added to buffered solutions (pH 2.0 to pH 9.5) of RMβCD, HPβCD, γ-CD, or SBEβCD at 0%, 5%, and 10% (*w*/*v*), incubated at 23 ± 1 °C, and sampled over time for UPLC quantification of lactone and hydroxyacid forms, and rate constants for the forward and reverse reaction were calculated. Phase solubility studies were also conducted to further characterize equilibrium behavior in aqueous CD systems. **Results**: The lactone form was most stable at a pH of 4.5, while the hydroxyacid form prevailed at a pH ≥ 7. γ-CD and HPβCD accelerated lactone hydrolysis for both statins, whereas RMβCD exerted a stabilizing effect. Increasing the CD concentration from 5% to 10% provided minimal additional stabilization. **Conclusions**: These findings highlight that the precise control of the pH, an appropriate cyclodextrin choice, and the selection of the statin form are critical to developing chemically stable eye drops.

## 1. Introduction

Age-related macular degeneration (AMD) is the leading cause of vision loss and blindness in the elderly population. AMD is classified into two types: dry and wet AMD [1]. The dry form of AMD begins when drusen, deposits of proteins and lipids, build up between, for example, the Bruch membrane and the retinal pigment epithelium (RPE) [2]. As drusen build up, the disease can progress to the wet form of AMD, where abnormal blood vessels grow in the back of the eye and damage the macula. Recently, faricimab (Vabysmo^®^) has been approved for the treatment of wet AMD, in addition to the current first-line treatments, which include ranibizumab (Lucentis^®^) and aflibercept (Eylea^®^), all of which have to be administered via intraocular injections. A major concern with these injections is the need for frequent administration (e.g., every 2 months in the best scenario) and the associated risks of retinal detachment, pain, and hemorrhage. These can lead to low compliance among patients, especially since the AMD treatment may be a lifelong process [3]. As an alternative, drugs formulated as eye drops could be a more suitable and convenient treatment option. Unfortunately, no effective topical treatments exist for the dry form of AMD. One possible strategy to tackle this disease is to target drusen deposits, which might prevent disease progression. Several studies have reported that oral statin therapy can slow disease progression [4,5,6,7,8]. This may be explained by the ability of statins to inhibit cholesterol synthesis in the retina.

**Figure 1:**
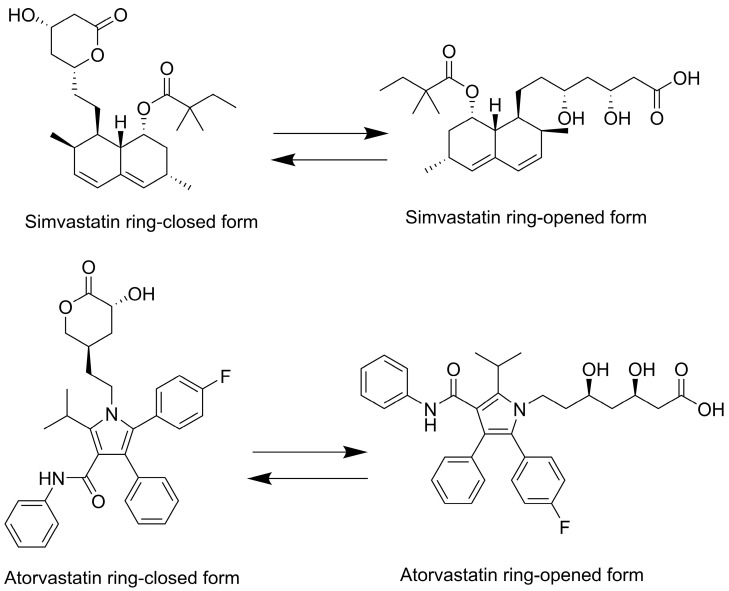
Two-dimensional chemical structures of simvastatin and atorvastatin. Both compounds are depicted in their lactone (prodrug) and acid (active) forms.

Statins such as simvastatin and atorvastatin can exist in two primary forms: the ring-closed lactone (inactive prodrug form) and the ring-opened hydroxyacid (active form), see Figure 1 [9]. Under physiological and alkaline pH conditions, statin lactones tend to hydrolyze to their hydroxyacid forms. Once the ring opens, the conversion of the hydroxyacid back to the lactone is negligible [10]. The topical ocular application of statins has shown promising benefits for ocular diseases such as AMD [5,11,12,13,14,15,16,17,18,19]. Building on these findings, formulation scientists have devoted their efforts to preparing stable topical statin delivery systems, such as nanoparticles [12,15], contact lenses [11], and micelles [14]. Simvastatin-loaded TPGS micelles have been developed with the goal of treating diseases in the posterior segment of the eye. Ex vivo porcine studies demonstrated some evidence of the conjunctival and scleral permeation of simvastatin [14]. In another study, eye drops containing solid lipid nanoparticles increased the bioavailability of atorvastatin in the vitreous humor by up to 48 times compared with the expected bioavailability of the oral administration of 40 mg of the drug [12]. Ex vivo studies of atorvastatin (molecular weight of 1155.3 g/mol and log P of 6.36) loaded into contact lenses demonstrated an improved accumulation in the sclera, with a lesser extent in the cornea. However, atorvastatin did not penetrate further, and this can be associated with its high hydrophobicity and large molecular weight [13]. A subsequent study replaced atorvastatin with pravastatin (molecular weight of 446.5 g/mol and log P of −0.23) in contact lenses. In vivo and ex vivo data showed that pravastatin can permeate through the sclera but not the cornea, and the drug accumulated in both tissues, suggesting that smaller, more hydrophilic statins exhibit distinct ocular transport characteristics [11].

Despite these encouraging findings, the physicochemical properties of statins, along with the complexity of the ocular barriers, pose significant challenges in achieving effective topical statin delivery, specifically to the back of the eye. Stabilizing the lactone form or controlling the lactone–acid ratio is critical for pharmaceutical stability, bioavailability, and efficacy. By elucidating the kinetics of lactone–acid interconversion and the factors that control this equilibrium, researchers can develop more stable ocular statin formulations. Key formulation considerations include (1) the limited aqueous solubility of hydrophobic statins (e.g., atorvastatin and simvastatin), (2) the chemical instability of statins in aqueous media, particularly the lactone–acid equilibrium, and (3) the predominant statin form (lactone vs. acid) and its impact on the corneal or conjunctiva–scleral absorption pathways reaching the posterior segment of the eye.

Cyclodextrins (CDs) are cyclic oligosaccharides. They are formed by the enzymatic degradation of starch and are composed of six (αCD), seven (βCD), or eight (γCD) glucose units. CDs form inclusion complexes with lipophilic drugs, a process where lipophilic drug moieties enter the central cavities of the CD molecules, leading to an increase in the solubility and bioavailability of aqueous solutions of these drugs. Furthermore, the complexation of drugs with CDs promotes their permeation through biological membranes [20]. CD-based eye drops can deliver therapeutic drug concentrations, such as dexamethasone, to the back of the eye [21,22,23]. However, statin–CD complexes or aggregates in aqueous eye drops for treating the posterior segment diseases have not been described. Both simvastatin and atorvastatin are lipophilic statins classified as Biopharmaceutics Classification System (BCS) class II drugs, characterized by a high permeability and low solubility [24,25]. Simvastatin and atorvastatin were chosen as representative statins based on their high clinical relevance and physicochemical properties. These drugs are the most prescribed statins [26,27], providing a well-established systemic safety profile, as well as a strong translational rationale. Both simvastatin and atorvastatin are considered lipophilic statins compared with hydrophilic ones, such as pravastatin and rosuvastatin [28]. Lipophilic statins can be preferred candidates for complexation with cyclodextrins.

While it is necessary to increase their solubility, it should not compromise the drug’s chemical stability and its subsequent absorption by the targeted tissue. For example, the lactone form may be more relevant for corneal permeation because of its higher lipophilicity and different transport mechanisms [29]. Thus, strategies aimed at improving solubility must also preserve the lactone form.

One effective strategy is the drug complexation with CDs, which has been successful in increasing the solubility of many drugs but also preserving their stability [30]. For example, the complexation of lovastatin or simvastatin with native βCD has been reported to enhance their aqueous solubility by 5-fold or 8-fold, respectively [31]. RMβCD forms even more stable complexes with both statins compared with native βCD [32]. It was suggested that this complexation might result in surface-active CD–statin complexes, particularly in the case of RMβCD, which may further contribute to the enhanced solubility observed [33]. Furthermore, HPβCD was shown to significantly enhance the solubility of simvastatin [34], while the solubility of atorvastatin was improved by βCD [35].

An important study in this context was reported by F. Ungaro et al. [36], who explored the enhancement of simvastatin solubility through complexation with 2-hydroxypropyl-β-cyclodextrin (HPβCD) and evaluated its impact on the drug’s chemical stability in aqueous solutions. However, it should be noted that while their paper reports the molecular weight of HPβCD as 14,598 Da, the typical molecular weight of HPβCD is between 1135 and 1600 Da, depending on the molar substitution based on both the supplier’s specifications (Sigma-Aldrich (St. Louis, MO, USA), Products No. 332607, No. 332593, and No. 389145) and the literature [37]. Consequently, any calculations (e.g., molar concentrations) based on 14,598 Da may have been off by nearly an order of magnitude if the standard form of HPβCD was used. Assuming that this was a typographical error and the MW of the HPβCD used was approximately 1460 Da, the concentration of the CD used was approximately 1% *w*/*v*.

In the context of ocular drug delivery, forming conventional simvastatin–CD inclusion complexes (i.e., dissolved drug–CD complexes) can enhance the solubility, but this low CD concentration may not be sufficient due to the very short residence time of dissolved water-soluble molecules on the eye surface. Beyond acting as solubilizers, CDs can self-assemble to form nano-and micro-aggregates, creating fast-dissolving drug–CD complex nanoparticles capable of delivering drugs to the eye in a more efficient way compared with conventional inclusion complexes. Upon application, these aggregates rapidly disassemble into nanoparticles with a longer residence time on the ocular surface due to the adhesion interaction with the mucus layer [21,38]. While CDs themselves cannot permeate ocular tissues, this mechanism mitigates rapid precorneal clearance, allowing the drug to be released in close proximity to ocular membranes. Consequently, a more comprehensive study exploring higher CD concentrations (e.g., 5–15% *w*/*v*), various pH values, and cyclodextrin types is needed to fully evaluate their potential as effective ocular delivery systems.

Our previous preliminary phase solubility studies did not clearly determine which statin form (lactone or hydroxyacid) predominates in the presence of various CD-buffered aqueous solutions (HPβCD, RMβCD, SBEβCD, and γCD). Therefore, prior to eye drop formulation studies, a more comprehensive investigation of chemical degradation is essential. Understanding the chemistry of CD–statin drug complexes in aqueous eye drops and how this chemistry influences the absorption of both forms into the back of the eye is crucial. Given that the CDs are not acting only as solubilizers but potentially also as drug carriers, the primary objective was to elucidate the influence of selected CDs at concentrations of 5% (*w*/*v*) and 10% (*w*/*v*) (necessary to form aggregates) on the equilibrium dynamics between the lactone and hydroxyacid forms of selected statins across varying pH conditions (2, 4.5, 7, 9.5). The secondary objective was to establish the formulation conditions necessary to develop stable CD-based eye drops incorporating simvastatin or atorvastatin, specifically determining the more stable statin form, the optimal CD concentration, and the appropriate pH range.

## 2. Materials and Methods

### 2.1. Materials

Simvastatin (SVT), [(1*S*,3*R*,7*S*,8*S*,8*aR*)-8-[2-[(2*R*,4*R*)-4-hydroxy-6-oxooxan-2-yl]ethyl]-3,7-dimethyl-1,2,3,7,8,8*a*-hexahydronaphthalen-1-yl] 2,2-dimethylbutanoate, molecular weight of 418.57 g/mol, practically insoluble in water [39], predicted pKa of 13.5 (SciFinder accessed on 3 October 2023), was purchased from Fluorochem Ltd. (Hadfield, UK). Simvastatin hydroxyacid (SVA), (3*R*,5*R*)-7-[(1*S*,2*S*,6*R*,8*S*,8*aR*)-8-(2,2-dimethylbutanoyloxy)-2,6-dimethyl-1,2,6,7,8,8*a*-hexahydronaphthalen-1-yl]-3,5-dihydroxyheptanoic acid, molecular weight of 436.6 g/mol, predicted pKa of 4.3 (SciFinder accessed on 3 October 2023), was also purchased from Fluorochem Ltd. (Hadfield, UK). Atorvastatin and atorvastatin lactone were purchased from Tokyo Chemical Industry Co., Ltd. (Zwijndrecht, Belgium). 2-Hydroxypropyl-β-cyclodextrin (HPβCD), molecular weight 1400 g/mol, randomly methylated-β-cyclodextrin (RMβCD), molecular weight 1310 g/mol, and γ-cyclodextrin (γCD), molecular weight 1297 g/mol, were purchased from Wacker Chemie AG (Munich, Germany). Captisol^®^ (sulfobutylether-β-cyclodextrin sodium salt (SBEΒCD)), molecular weight 2163 g/mol, was purchased from CyDex, Inc. (San Diego, CA, USA). Milli-Q water (Millipore, Billerica, MA, USA) was used to prepare the mobile phases and CD solutions. All other chemicals used were of analytical grade.

### 2.2. Methods

#### 2.2.1. Simultaneous Quantitative Determination of Simvastatin and Simvastatin Acid or Atorvastatin Acid and Atorvastatin Lactone

Quantitative determination of simvastatin was performed using a reversed-phase high-performance liquid chromatography (HPLC) system (ACQUITY ARC UHPLC Waters, MassLynx Software V4.2). The method described here was modified from a previously published method [14]. The system was operated at a flow rate of 0.5 mL/min and a temperature of 20.4 °C. The column used was an XBridge™ Premier BEH C18 (2.5 µm, 4.6 × 50 mm, Waters Technologies Ireland Ltd., Wexford, Ireland). Isocratic mobile phase was applied to elute the drug: (A) 0.1% (*v*/*v*) formic acid, (B) acetonitrile (35:65, *v*/*v*). The wavelength was 240 nm for simvastatin and 246 nm for atorvastatin. The injection volume was 10 µL. Method linearity for each statin form was confirmed between 1 and 10 µg/mL (r^2^ ≥ 0.999).

#### 2.2.2. The Determination of the Water Content

The moisture contents of the powders of HPβCD, SBEβCD, RMβCD, and γCD were determined using a moisture analyzer (A&D MX-50) (Tokyo, Japan). CD powder (1 g) was evenly distributed over an aluminum pan to achieve homogenous drying (*n* = 3).

#### 2.2.3. Phase Solubility Studies of Simvastatin and Atorvastatin

##### Aqueous Solubility of Simvastatin and Atorvastatin

An excess amount of simvastatin lactone or atorvastatin acid powder was added to 3 mL of MQ water or buffer. The suspensions formed were placed in a shaker (LAB-LINE^®^ ORBIT ENVIRON-SHAKER, LAB-LINE INSTRUMENTS, Inc., Melrose Park, IL, USA) for 5 days. The equilibration was achieved at 23 ± 1 °C under constant agitation at 260 RPM. The saturated mixture was filtered through 0.45 µm filters (PHENEX PTFE, Phenomenex, Torrance, CA, USA). The filtrate was then analyzed using HPLC. All experiments were carried out in triplicate unless otherwise stated.

##### Preliminary Phase Solubility Study of Simvastatin in Phosphate Buffer (0.1 M, pH 7.01)

Phase solubility studies were conducted according to the method reported by Higuchi and Connors [40]. An excess amount of simvastatin was added to aqueous solutions of 0–15% (*w*/*v*) HPβCD, RMβCD, and γCD. These simvastatin–CD suspensions were prepared in phosphate buffer (0.1 M, pH 7.01). Moisture content was determined, and the weight of the CD powders was adjusted accordingly. Upon mixing, each suspension underwent 2 min vortexing and were subsequently placed in a shaker (LAB-LINE^®^ ORBIT ENVIRON-SHAKER, LAB-LINE INSTRUMENTS, Inc., Melrose Park, IL, USA). Equilibration was achieved at 23 ± 1 °C under constant agitation at 120 RPM for 20 days. The saturated mixtures were filtered using 0.45 µm filters (PHENEX PTFE, Phenomenex, Torrance, CA, USA). The filtrates were then diluted 10-fold or as appropriate with 65% acetonitrile to bring the analyte concentration within the HPLC linearity range. During the filtration step, only the clear upper portion of the suspension was collected after equilibration to avoid disturbing any undissolved drug. All experiments were conducted in triplicate unless otherwise stated.

##### Phase Solubility Studies of Simvastatin in Citrate Buffer (0.1 M, pH 4.82) and Atorvastatin in Phosphate Buffer (0.1 M, pH 7.01)

The phase solubility studies were performed as described in Section Preliminary Phase Solubility Study of Simvastatin in Phosphate Buffer (0.1 M, pH 7.01) but using only HPβCD and γCD (HPβCD and γCD were selected for further studies due to their superior ocular tolerability compared with RMβCD). Suspensions were agitated at 260 RPM for 5 days and protected from light throughout the equilibration period.

#### 2.2.4. Chemical Stability of Statins in Aqueous CD Solutions

##### Buffer Preparations

A universal buffer (0.15 M) was prepared by dissolving appropriate amounts of citric acid (Mw 192.12 g/mol), sodium phosphate dibasic heptahydrate (Mw 268.07 g/mol), and boric acid (Mw 61.83 g/mol) in Milli-Q (MQ) water. Concentrated NaOH or HCL was then used to prepare solutions with pH values of 2, 4.5, 7, and 9.5.

##### Preparation of Cyclodextrin Aqueous Solutions

First, the CD moisture content was determined in triplicate using a moisture analyzer (A&D MX-50) (Tokyo, Japan) and then considered in the subsequent mass calculations for the preparation of CD aqueous solutions.

Second, aqueous solutions of each CD were prepared in buffered solutions at concentrations of 5% (*w*/*v*) and 10% (*w*/*v*).

##### Preparation of Statin Stock Solutions

Stock solutions of 1 mg/ mL of simvastatin or atorvastatin were prepared in ethanol–water (50:50, *v*/*v*) and kept at 4 ± 1 °C until used.

##### Addition of Drug to Buffered Solutions with and Without Cyclodextrin

A 100 µL volume of the stock solution (1 mg/mL) was added to 10 mL solutions containing 0%, 5% (*w*/*v*), and 10% (*w*/*v*) of RMβCD, HPβCD, γCD, and SBEβCD at various pH values. The solutions were left at room temperature (23 ± 1 °C), and sampling was performed every 48 h.

##### Ring Opening–Ring Closed in Buffered Solutions

Stock solutions of the lactone and acid forms of simvastatin and atorvastatin were prepared in ethanol–water (50:50, *v*/*v*). Defined aliquots of each stock were added, in duplicate (simvastatin only), to buffered solutions (pH 2, 4.5, 7, and 9.5). Samples were incubated at 23 ± 1 °C, and the chromatographic peak areas corresponding to the lactone and acid forms were quantified at predetermined intervals.

## 3. Results

### 3.1. The Effect of pH on the Ring-Opened/Closed Equilibrium of Simvastatin and Atorvastatin Without Cyclodextrin

**Figure 2:**
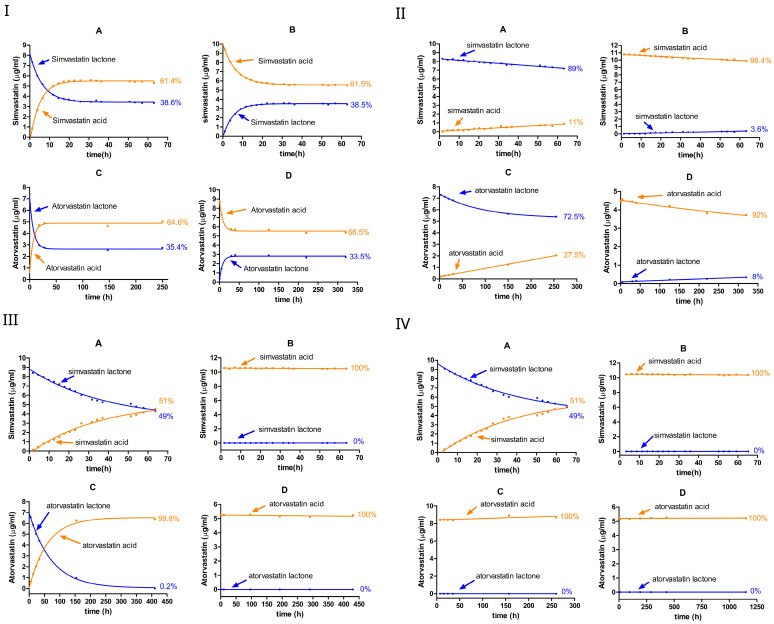
The equilibrium between the lactone and hydroxyacid forms of simvastatin and atorvastatin in the 0% CD-buffered aqueous solution at various pH levels: pH 2 (**I**), pH 4.5 (**II**), pH 7 (**III**), and pH 9.5 (**IV**). A, simvastatin lactone; B, simvastatin acid; C, atorvastatin lactone; and D, atorvastatin acid. Blue dots represent the lactone form, while orange dots represent the hydroxyacid form.

We investigated the equilibrium dynamics between the lactone (ring-closed) and hydroxyacid (ring-opened) forms of simvastatin and atorvastatin in buffered solutions at various pH values, as shown in Figure 2 and Table 1. The conversion was monitored over time, starting from either the lactone or hydroxyacid form for each statin. In the case of simvastatin, the equilibrium was studied starting from the lactone form (Figure 2(IA–IVA)) or hydroxyacid form (Figure 2(IB–IVB)). Likewise, for atorvastatin, the conversion was examined starting from the lactone form (Figure 2(IC–IVC)) or from the hydroxyacid form (Figure 2(ID–IVD)).

At pH 2, the rate constants for both forward (lactone → acid) and reverse (acid → lactone) reactions were comparably high, establishing an equilibrium within approximately 24 h and yielding similar lactone-to-acid ratios irrespective of whether the starting form was initially ring-closed or ring-opened (Figure 2I, Table 1). For simvastatin, the forward rate constant ($k_{f})$ for the ring-closed form (lactone hydrolysis) and the reverse rate constant ${(k}_{r})$ for the ring-opened form (lactonization) were nearly identical at around 0.1 h^−1^. Likewise, the rate constants for the acid formation and acid loss were closely matched at nearly 0.06 h^−1^. The similar values obtained confirm the reversible chemistry in this reaction. Similarly, the ring-opening and ring-closing reactions of atorvastatin showed reversibility and proceeded more rapidly compared with those of simvastatin.

At equilibrium, simvastatin exhibited a mixture of around 38% lactone and 62% acid, whereas atorvastatin maintains 33–35% lactone and 67–65% acid, regardless of whether the reaction started from the lactone or acid form. The calculated rate constants in Table 1 confirm this observation, where the $k_{f}$ of the ring-closed form was higher than the $k_{f}$ of the ring-opened form, which means that overall $k_{f}>k_{r}$, thus favoring the ring-opened (the acid) form. The data fit a pseudo-first-order kinetic model in both directions; however, the rate constants tended to be lower when the process started from the ring-opened form.

The overall pace of the interconversion markedly diminished as the pH increased to 4.5. Compared with pH 2, the hydrolysis rate constants of both simvastatin and atorvastatin were approximately 33 to 46 fold lower, respectively. Similarly to pH 2, $k_{f}>k_{r}$, which favors acid formation. The degradation kinetics of the lactone form were comparable between atorvastatin and simvastatin, with no significant difference observed in the forward rate constants for the ring-closed forms of the two compounds.

The degradation of simvastatin and atorvastatin was much faster at pH 2 than at pH 4.5. The lactone ring of atorvastatin appears to be more susceptible to acid-catalyzed hydrolysis, where it shows higher forward rates relative to simvastatin. At pH 2, the equilibrium between the two forms was reached within just a few hours, resulting in similar ring-closed/ring-opened ratios, regardless of the initial form (i.e., the acid or lactone). In contrast, the equilibrium appears to take a longer time to be reached at pH 4.5.

When the pH increased to 7 or 9.5, the interconversion of lactone⇌acid seemed to diminish completely for both simvastatin and atorvastatin, favoring the ring-opened (acid) form. For simvastatin, the hydrolysis rate constant was similar at pH 7 and pH 9.5. This was approximately four times higher than the values obtained at pH 4.5, but approximately seven times lower than those obtained at pH 2. Meanwhile, when starting from the ring-opened form, simvastatin remained stable at both pH 7 and pH 9.5, with a negligible rate constant—the rate constant was below the detection limit compared with those obtained at other pH values. A similar trend was observed for atorvastatin, with a complete conversion to the ring-opened form within 7 h at pH 9.5.

Overall, across the investigated pH levels, the lactone–acid equilibrium was pH-dependent for both simvastatin and atorvastatin. At pH 2, the interconversion was rapid in both directions for both statins, reaching an equilibrium mixture of lactone and acid forms within a few hours. Increasing the pH to 4.5 significantly reduced the rate constants and prolonged the time required to establish an equilibrium. Under neutral and alkaline conditions (pH 7 and pH 9.5, respectively), the ring-opened form (acid form) was predominant, as the equilibrium effectively shifted to favor the acid form.

Moreover, the acid forms of simvastatin and atorvastatin possess carboxylic acid moieties with pK_a_ values near 4.3. At physiological and alkaline pH values (e.g., pH 7.0 and 9.5), the ionization of the carboxyl group is favored, resulting in the predominance of the ring-opened form (lactone ring formation is not favored under these conditions). In contrast, under acidic conditions (pH < pK_a_), the carboxyl group remains mostly non-ionized, favoring the ring-closed form.

Among statin drugs, the pharmacologically active form is the hydroxyacid form. Consequently, they are administered in this open-ring form. However, simvastatin and lovastatin are exceptions; they are administered as inactive lactone prodrugs, which undergo in vivo hydrolysis to yield the active hydroxyacid metabolite [41]. For the statins investigated in this study, simvastatin and atorvastatin, the acid-catalyzed interconversion can be considered a reversible process, whereas the base-catalyzed reaction appears to be irreversible, agreeing with a previous work [42].

**Figure 3:**
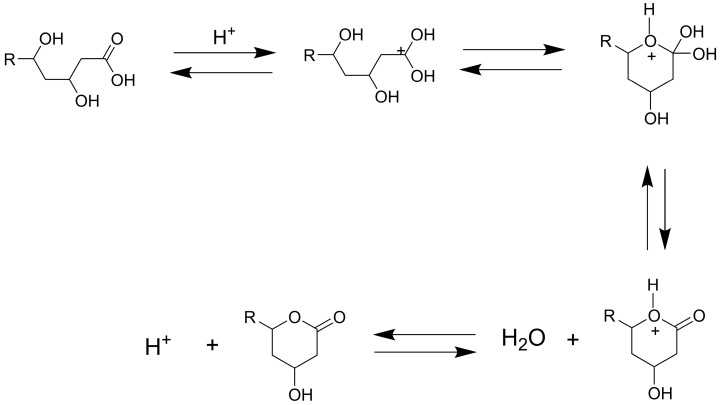
Acid-catalyzed ring-closing and ring-opening mechanisms of atorvastatin, involving the reversible interconversion between the lactone and hydroxyacid forms; adapted from [42].

The ring-closing and ring-opening mechanisms of atorvastatin were experimentally established by [42]. For the acid-catalyzed reaction, the acid-to-lactone reaction starts with the protonation of the carbonyl oxygen, followed by the intramolecular nucleophilic attack by the hydroxyl group to produce a tetrahedral intermediate, which subsequently collapses to expel water and form the lactone ring. This reaction is reversible; the lactone-to-acid reaction proceeds with water serving as the nucleophile to attack the protonated lactone carbonyl to form a tetrahedral intermediate, ultimately regenerating the hydroxyacid form (Figure 3) [42].

In the base-catalyzed reaction, the conversion of the lactone to the acid form can be considered irreversible, proceeding through the nucleophilic attack by the hydroxide ion, which facilitates acyl bond cleavage via a tetrahedral intermediate [42]. The density functional theory (DFT) calculations further support these ring-closing and ring-opening mechanisms in atorvastatin [43]. For example, under basic conditions, the hydrolysis reaction requires <10 kcal mol^−1^, but lactonization would take approximately 28 kcal mol^−1^.

**Figure 4:**
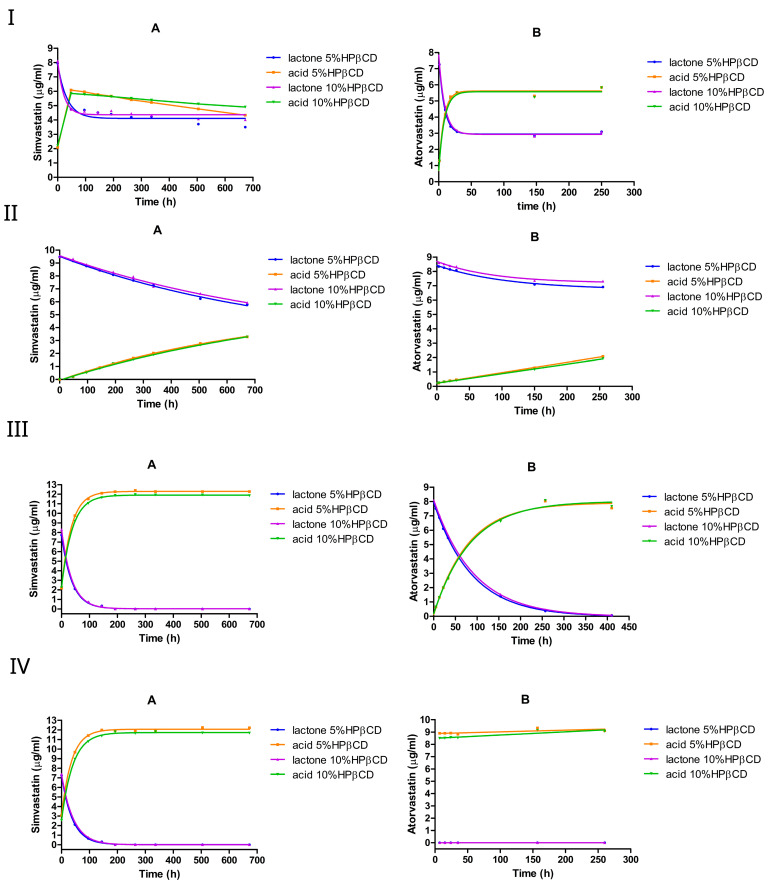
The equilibrium between the lactone and hydroxyacid forms of simvastatin and atorvastatin in the presence of HPβCD at pH 2 (**I**), pH 4.5 (**II**), pH 7 (**III**), and pH 9.5 (**IV**). A is simvastatin and B is atorvastatin.

**Figure 6:**
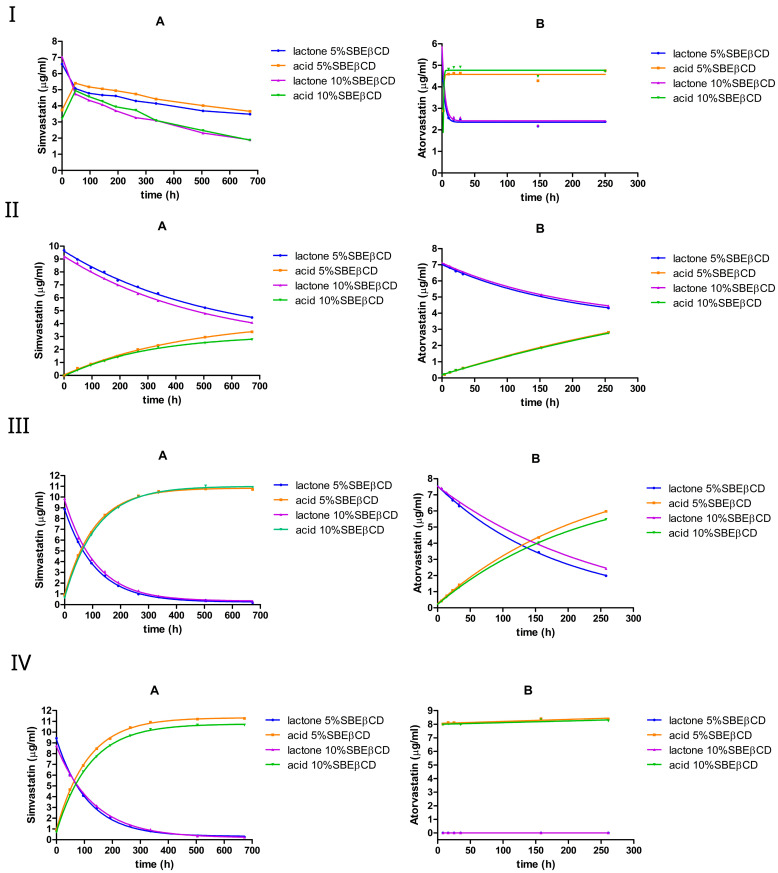
The equilibrium between the lactone and hydroxyacid forms of simvastatin and atorvastatin in the presence of SBEβCD at pH 2 (**I**), pH 4.5 (**II**), pH 7 (**III**), and pH 9.5 (**IV**). A is simvastatin and B is atorvastatin.

The interconversion between the lactone and hydroxyacid forms of simvastatin follows acid- and base-catalyzed mechanisms similar to those observed in atorvastatin. Under acidic conditions, these reactions are reversible, while under basic conditions, lactone hydrolysis is strongly favored, leading to the hydroxyacid as the dominant form. In addition to lactone hydrolysis, the 2,2-dimethyl-butanoic acid ester moiety of simvastatin presents another potential hydrolysis site. However, due to steric hindrance, the ester hydrolysis is expected to be significantly slower compared with lactone hydrolysis, and it may be negligible under these conditions. However, Figure 4 and Figure 6(IA) suggest the further degradation of the hydroxyacid form in the presence of SBEβCD or HPβCD, which may indicate gradual ester hydrolysis or other degradation pathways.

In general, these findings are consistent with previous studies [43,44,45,46,47]. For example, the simvastatin degradation is pH-dependent, with a significant increase in degradation rates as the pH increases from 5 to 8 [44]. This suggests that simvastatin tends to be more stable under mildly acidic conditions (i.e., pH 4–5) than under more alkaline conditions. This trend is further supported by the rate constants obtained in this study, where the forward hydrolysis rate (*k_f_*) was significantly lower at 0.003 h^−1^ at pH 4.5 compared with approximately 0.01 h^−1^ at pH 7 or pH 9.5. In another study, the simvastatin lactone-to-acid conversion was found to be faster in gastric fluids than in intestinal fluids [45]. This observation aligns with our results, which demonstrated that both simvastatin and atorvastatin undergo a more rapid hydrolysis under strongly acidic conditions (i.e., pH 2) than at higher pH levels (Table 1). Furthermore, the same study suggested the interconversion between the lactone and the acid (lactone⇌acid) upon the incubation in gastric media [45], which also agrees with our results that the equilibrium is established within a few hours with similar lactone/acid ratios, regardless of the initial form (Figure 2I, Table 1). Interestingly, the simvastatin acid-to-lactone conversion was found to be negligible in intestinal fluids [45]. This is also consistent with our results, where no detectable acid-to-lactone conversion was observed at pH 7 or above for either statin. These results collectively highlight the pH dependence of the statin interconversion, with acidic conditions favoring both hydrolysis and reversibility, whereas neutral and alkaline conditions strongly favor the hydroxyacid form. This is also in agreement with [42], which observed an equilibrium favoring the atorvastatin acid form at pH < 6, whereas no equilibrium was detected at pH > 6, and the acid form predominated. The acid form of atorvastatin was also demonstrated to be much more stable than the lactone form under alkaline conditions [43]. This agrees with our results, where the lactone form underwent a full conversion to the acid form within 7 h at pH 9.5, whereas no acid-to-lactone conversion was detected at pH 7 or 9.5.

Based on our findings and previous reports, it can be concluded that under acidic conditions (particularly near pH 2), the lactone–acid interconversion occurs rapidly, reaching an equilibrium within a few hours, with the acid form being favored at around 60–65%, regardless of the statin’s initial form. In contrast, under neutral or alkaline pH conditions, this equilibrium is significantly diminished, and the conversion from the lactone to acid appears to be irreversible, with acid being the predominant form.

This ring-closed/opened study was necessary to establish the baseline solubility and chemical stability of each statin in the absence of CDs. The next question was whether the inclusion complexation with CDs can simultaneously (i) enhance the solubility of simvastatin and atorvastatin and (ii) slow down lactone hydrolysis, thereby preserving the drug throughout the formulation’s shelf-life.

### 3.2. Effects of Various Cyclodextrins on the Stability of Lactone in Simvastatin and Atorvastatin

#### 3.2.1. The Effect of 5% or 10% (*w*/*v*) HPβCD 

We investigated the effect of HPβCD on lactone hydrolysis using two statin drugs, simvastatin and atorvastatin, at two different concentrations (5% *w*/*v* and 10% *w*/*v*), as shown in Figure 4 and Table 2. The conversion was monitored over time, starting from the lactone form.

To characterize the forward hydrolysis of simvastatin and atorvastatin from their lactone to acid forms, we monitored both the loss of the lactone (ring-closed form) and the accumulation of the acid (ring-opened form) over time. Because no measurable reverse reaction (acid → lactone) was observed, these two datasets describe the same forward reaction ($k_{f}$) from two perspectives: the disappearance of the lactone and the appearance of the acid (Table 3). To quantify the rate constant, we fitted the lactone and acid data separately to one-phase kinetics models.

At pH 2, both simvastatin and atorvastatin underwent rapid lactone hydrolysis, reaching an equilibrium within a few hours, favoring the acid form (Figure 4I). The addition of HPβCD at 5% or 10% (*w*/*v*) reduced the hydrolysis rate, especially of simvastatin, where the forward rate constant $k_{f}$ dropped by approximately 2–4 times compared with the medium without HPβCD. However, increasing the HPβCD concentration from 5% to 10% nearly doubled the forward rate constant of simvastatin, but slightly reduced the forward rate constant of atorvastatin (Table 2). Interestingly, HPβCD at both concentrations shifted the lactone/acid equilibrium from 38%:62% (no cyclodextrin) to approximately 45%:55 % for simvastatin, whereas it remained unchanged at approximately 35%:65% for atorvastatin. One factor contributing to this difference between the two drugs is the significantly stronger complex formation between simvastatin and HPβCD ($K_{1:1}\approx 3000$ $M^{-1}$ at pH 7 and $K_{1:1}\approx 270$ $M^{-1}$ at pH 4.82) compared with atorvastatin ($K_{1:1}\approx 164$ $M^{-1}$ at pH 7). Importantly, doubling the HPβCD concentration resulted in similar lactone/acid ratios at equilibrium for both drugs, indicating that adding more HPβCD does not affect the final mixture composition.

In the absence of HPβCD at pH 4.5, the forward rate constant $k_{f}$ of the lactone hydrolysis was approximately two times higher than the reverse rate constant $k_{r}$ for simvastatin and atorvastatin (Table 1), favoring hydrolysis over re-lactonization. However, the presence of HPβCD led to an approximately 4-fold decrease in lactone hydrolysis in simvastatin. The forward rate constant $k_{f}$ for simvastatin dropped by approximately 4-fold from 0.003 h^−1^ without cyclodextrin to 0.0008 h^−1^, while in atorvastatin, the rate dropped by 3-fold (Table 2). It is worth noting that doubling the HPβCD concentration (from 5% to 10%) had a negligible effect on the rate constants, as they remained very similar between the two cyclodextrin levels. For both simvastatin and atorvastatin, the lactone hydrolysis followed pseudo-first-order kinetics.

However, a different scenario was observed under neutral and alkaline conditions with simvastatin; at pH 7 and pH 9.5, introducing HPβCD at 5% or 10% (*w*/*v*) led to an approximately 2-fold increase in lactone hydrolysis. In contrast, HPβCD slightly reduced the conversion in atorvastatin at pH 7, although it still underwent a complete lactone-to-acid conversion within 7 h at pH 9.5. The hydrolysis reaction follows pseudo-first-order kinetics under neutral or alkaline pH conditions.

Overall, the influence of the HPβCD addition seems to differ between simvastatin and atorvastatin. As indicated in Table 2, HPβCD at 5% or 10% (*w*/*v*) reduced atorvastatin lactone hydrolysis rates at pH 2, pH 4.5, and pH 7. For simvastatin, HPβCD decreased the lactone hydrolysis rate under acidic conditions, but increased it under neutral and alkaline conditions. These findings align with those of previous studies [36,44], where HPβCD (∼1% *w*/*v* at pH 5.5) was reported to increase simvastatin lactone hydrolysis by approximately 2-fold [36], further supporting the faster lactone degradation observed in the presence of HPβCD in our study.

Nevertheless, two questions remain unresolved: (1) why does HPβCD exert a different influence on simvastatin compared with atorvastatin and (2) why does increasing the HPβCD concentration not further enhance the stabilization of simvastatin?

For example, at pH 7, simvastatin exhibits a substantially stronger complex formation with HPβCD ($K_{1:1}\approx 3000$ $M^{-1}$) compared with atorvastatin ($K_{1:1}\approx 164$ $M^{-1}$), implying that HPβCD should provide more protection to simvastatin. This might be true if the lactone ring is taking part in the complex formation with HPβCD. However, the 2,2-dimethyl-butanoic acid ester moiety of simvastatin, an additional hydrolysis site, is proposed to take part in the complexation, leaving the lactone ring to be only partially incorporated or to be completely unincorporated within the hydrophobic cavity of HPβCD [32,36]. Consequently, because simvastatin is susceptible to hydrolysis at both the lactone ring and the ester (with the lactone ring being more readily hydrolyzed), the preferential complexation with the ester could render the lactone ring more exposed to hydrolysis. This scenario could help explain why HPβCD does not confer as much protection as one might expect solely based on binding constants.

Moreover, if the simvastatin–HPβCD complexation process does not involve the lactone ring, then hydrolysis can proceed not only in the free drug but also in the drug bound to HPβCD. Interestingly, the stability constant between simvastatin and HPβCD at pH 7 is 3000 $M^{-1}$, whereas at pH 4.82 it is only 270 $M^{-1}$. Given the predominance of the open-ring (acid) form at pH 7 and the lactone form at pH 4.82 under our study conditions, this might suggest that the absence of the lactone ring promotes stronger binding to HPβCD. This observation is consistent with previous reports [36], which demonstrated that both the ring-closed and ring-opened forms of simvastatin exhibit comparable interactions with HPβCD; hence, even where 10% HPβCD results in a larger fraction of the drug being bound compared with 5%, hydrolysis can still proceed. This helps clarify why increasing the HPβCD concentration did not further enhance the chemical stability of simvastatin lactone.

#### 3.2.2. The Effect of 5% or 10% (*w*/*v*) RMβCD

**Figure 5:**
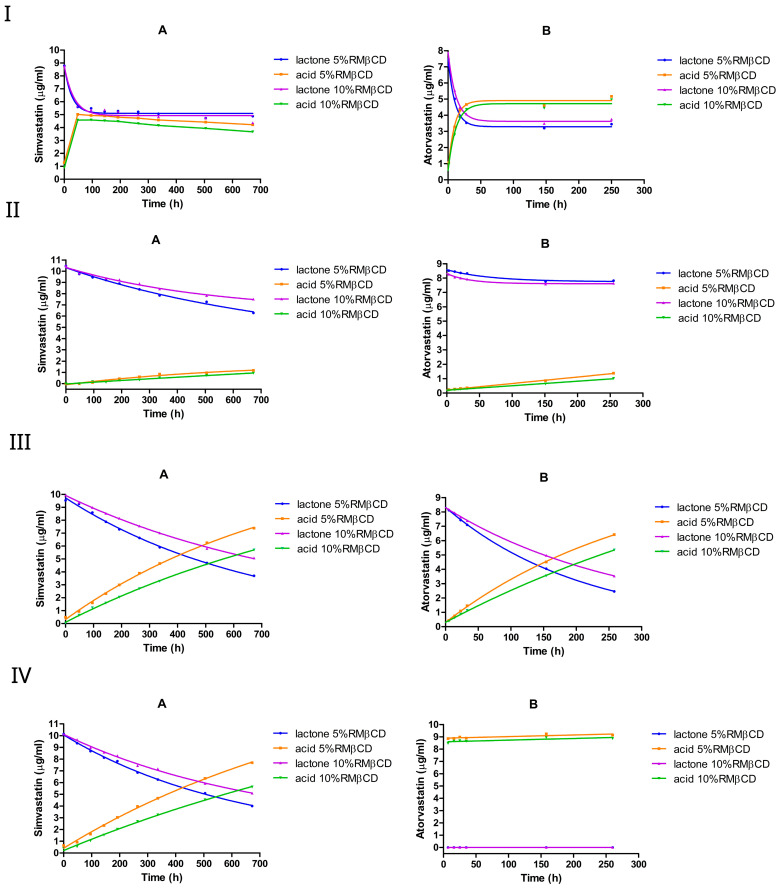
The equilibrium between the lactone and hydroxyacid forms of simvastatin and atorvastatin in the presence of RMβCD at pH 2 (**I**), pH 4.5 (**II**), pH 7 (**III**), and pH 9.5 (**IV**). A is simvastatin and B is atorvastatin.

At pH 2, both simvastatin and atorvastatin underwent rapid lactone hydrolysis in the presence of RMβCD, exhibiting a kinetic profile similar to that observed with HPβCD (Figure 5I). However, RMβCD decreased the hydrolysis rate by approximately threefold in simvastatin, from approximately 0.1 h^−1^ to around 0.03 h^−1^, and by approximately onefold in atorvastatin, from 0.14 h^−1^ to around 0.09 h^−1^.

Moreover, the addition of RMβCD significantly shifted the equilibrium composition towards the lactone form, particularly for simvastatin, where the equilibrium ratio of lactone/acid changed significantly from 38%:62% (without cyclodextrin) to approximately 55%:45%. In the case of atorvastatin, the equilibrium shifted slightly towards the lactone form, with the lactone/acid ratio moving from 35%:65% (without cyclodextrin) to approximately 43%:57%. Compared with HPβCD, RMβCD seems to exert a more pronounced stabilizing effect on the lactone form under strongly acidic conditions. Unlike HPβCD, increasing RMβCD from 5% to 10% *w*/*v* resulted in a lower hydrolysis rate constant in both drugs. Lactone in atorvastatin seemed to be degraded faster than in simvastatin, and both seemed to follow pseudo-first-order kinetics.

At pH 4.5, the lactone hydrolysis rate in the presence of RMβCD was slightly lower than that observed with HPβCD for simvastatin, whereas for atorvastatin, the hydrolysis rate was approximately 2–4-fold lower compared with the cyclodextrin-free medium, indicating a protective influence of RMβCD on the lactone form under mildly acidic conditions. Specifically, RMβCD decreased the lactone hydrolysis rate in simvastatin by approximately 4-fold, dropping from 0.003 h^−1^ without cyclodextrin to 0.0007 h^−1^ at 5% *w*/*v* and by 6-fold (to 0.0005 h^−1^) at 10% *w*/*v* (Table 4). In atorvastatin, on the other hand, the hydrolysis rate dropped by approximately 4-fold, from 0.003 h^−1^ to 0.0008 h^−1^ at 5% *w*/*v*, and by around 2-fold at 10% *w*/*v*, underscoring the more pronounced lactone-stabilizing effect of RMβCD in atorvastatin. Doubling the RMβCD concentration (from 5% to 10%) resulted in a slight decrease in the hydrolysis rate constants for simvastatin and a slight increase for atorvastatin; moreover, for both drugs, the lactone hydrolysis followed pseudo-first-order kinetics.

In contrast to HPβCD, the RMβCD addition resulted in a lower lactone hydrolysis rate in simvastatin at both pH 7 and pH 9.5. In atorvastatin, a lower lactone hydrolysis rate was also obtained at pH 7. Moreover, relative to a cyclodextrin-free medium, 10% RMβCD yielded a 14-fold decrease in the hydrolysis rate constant for simvastatin at pH 7 and pH 9.5. Likewise, an approximately 4-fold reduction in the rate constant was observed with atorvastatin at pH 7. Interestingly, increasing the RMβCD concentration from 5% to 10% (*w*/*v*) resulted in a slight drop in the hydrolysis rate constant in both drugs. However, atorvastatin lactone remained more susceptible to degradation compared with simvastatin under these conditions; atorvastatin underwent a complete lactone-to-acid conversion within 7 h at pH 9.5.

Overall, RMβCD tends to exert a more protective effect on the lactone of both simvastatin and atorvastatin compared with HPβCD or cyclodextrin-free media, especially under mild acidic conditions. One possible explanation for this difference is that, in randomly methylated β-cyclodextrin, approximately two-thirds of the hydroxyl groups are methylated. As a result, these modified groups can no longer act as hydrogen-bond donors. This modification also increases the hydrophobicity of the CD cavity, potentially resulting in stronger drug binding. This interpretation is consistent with the stability binding data, which revealed a notably stronger interaction between simvastatin and RMβCD ($K_{1:1}\approx 11300$ $M^{-1}$) than HPβCD ($K_{1:1}\approx 3000$ $M^{-1}$).

#### 3.2.3. The Effect of 5% or 10% (*w*/*v*) SBEβCD

At pH 2, both simvastatin and atorvastatin underwent a rapid interconversion in the presence of SBEβCD, reaching an equilibrium within a few hours, with the acid form being favored (Figure 6I). The addition of SBEβCD shifted the equilibrium composition towards the lactone form in simvastatin, where the equilibrium ratio of lactone/acid was 49%:51% compared with 38%:62% (no cyclodextrin). In the case of atorvastatin, the equilibrium shifted slightly towards the acid form, with the lactone/acid ratio moving from 35%:65% (no cyclodextrin) to approximately 33%:67%.

The calculated rate constants (Table 5) support this observation; the addition of SBEβCD reduced the hydrolysis rate constants by 3–5-fold in simvastatin, but increased them by approximately 3-fold in the case of atorvastatin. However, after reaching equilibrium, further degradation seemed to occur only with simvastatin, especially when SBEβCD was increased from 5% to 10% *w*/*v*. This additional degradation likely arises from the hydrolysis of the 2,2-dimethyl-butanoic acid ester moiety of simvastatin. This was most pronounced with SBEβCD at pH 2 (Figure 6(IA)). It is likely that the negatively charged sulfobutyl substituents of SBEβCD can alter the binding orientation of the drug’s ester group.

At pH 4.5, the simvastatin lactone hydrolysis seemed to proceed more rapidly in the presence of SBEβCD compared with HPβCD or RMβCD. The forward rate constant ($k_{f}$) was approximately 0.002 h^−1^, which is approximately three-fold higher than those observed with HPβCD and RMβCD, but it is still approximately one-fold lower than the rate constant observed without cyclodextrin. The same trend was also observed with atorvastatin. Doubling the SBEβCD concentration (from 5% to 10%) had a negligible effect on the hydrolysis rate constants of both drugs.

At pH 7, similar to RMβCD, the addition of SBEβCD resulted in a reduction in lactone hydrolysis in both statins; specifically, the forward rate constant ($k_{f}$) decreased by approximately 2-fold in simvastatin and 3-fold in atorvastatin. For simvastatin, the forward rate constant was approximately three times lower than that obtained with HPβCD but remained approximately nine times higher than that obtained with RMβCD. In atorvastatin, the forward rate constant was approximately two times lower than that of HPβCD but was very similar to that observed with RMβCD. However, simvastatin lactone appeared to be more susceptible to degradation than atorvastatin lactone under these conditions. This may be similar to HPβCD: if the complexation is taking place with the 2,2-dimethyl-butanoic acid ester moiety of simvastatin, then the lactone ring remains susceptible to ring opening. Increasing the SBEβCD concentration from 5% to 10% (*w*/*v*) resulted in a small drop in the hydrolysis rate constant of atorvastatin but did not seem to have a significant effect on simvastatin. Similar rate constants were observed for simvastatin at pH 9.5, and a complete lactone-to-acid conversion was observed in atorvastatin within a few hours.

SBEβCD generally lowered the hydrolysis rates of simvastatin across the tested pH values. A similar effect was observed with atorvastatin, but only at mild-to-moderate pH ranges.

#### 3.2.4. The Effect of 5% or 10% (*w*/*v*) γCD

**Figure 7:**
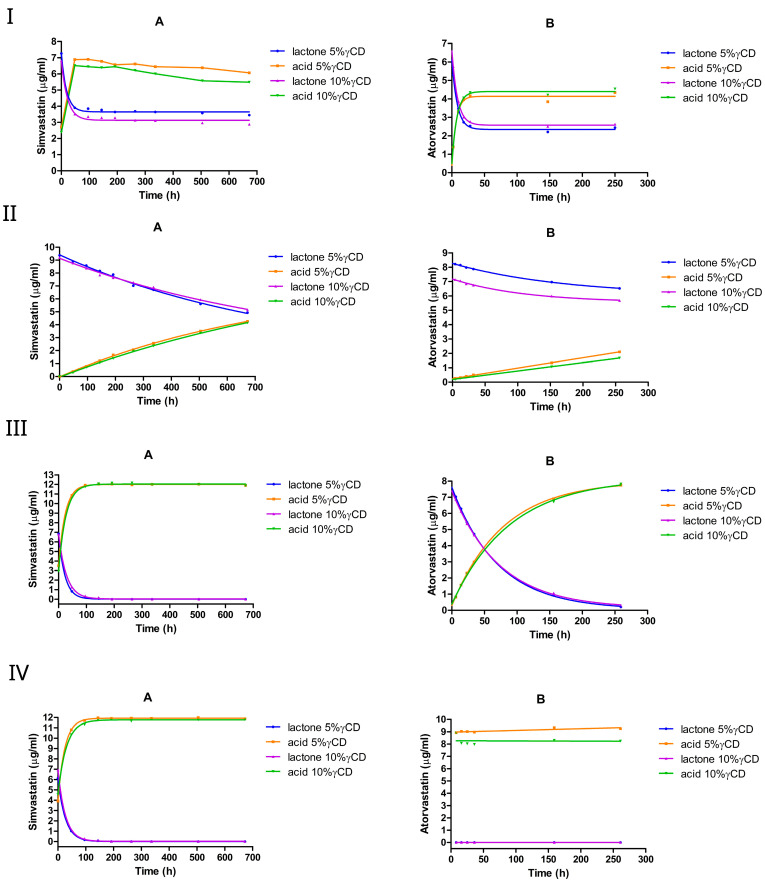
The equilibrium between the lactone and hydroxyacid forms of simvastatin and atorvastatin in the presence of γCD at pH 2 (**I**), pH 4.5 (**II**), pH 7 (**III**), and pH 9.5 (**IV**). A, simvastatin; B, atorvastatin.

Like other CDs, both simvastatin and atorvastatin underwent a rapid interconversion in the presence of γCD at pH 2, reaching an equilibrium within a few hours, with the acid form being favored (Figure 7I).

The corresponding hydrolysis rate constants declined by approximately two-fold in simvastatin and one-fold in atorvastatin. In simvastatin, the addition of γCD slightly shifted the equilibrium toward the acid form, with the lactone/acid ratio changing from 38%:62% (in the absence of cyclodextrin) to 36%:64%. By contrast, in atorvastatin, the equilibrium shifted slightly toward the lactone form, with the lactone/acid ratio moving from 35%:65% (no cyclodextrin) to approximately 36%:64%. Furthermore, the hydrolysis rate constant of both drugs dropped slightly when the γCD concentration was increased from 5% to 10% *w*/*v*.

At pH 4.5, γCD slowed down the lactone hydrolysis in simvastatin, but increased the rate in atorvastatin. In simvastatin, the hydrolysis rate constant dropped by 3-fold at 5% *w*/*v* γCD and 4-fold at 10% *w*/*v* γCD. In atorvastatin, it increased by approximately 2-fold with 5% *w*/*v* γCD and 3-fold with 10% *w*/*v* γCD. Doubling the γCD concentration from 5% to 10% resulted in a one-fold decrease in simvastatin hydrolysis, but accelerated hydrolysis in atorvastatin. At pH 7 and pH 9.5, γCD showed an approximately 3-fold faster hydrolysis rate in simvastatin, with the constant rate increasing from approximately 0.014 h^−1^ to approximately 0.044 h^−1^; conversely, it had a negligible impact on atorvastatin at pH 7 (Table 6).

Overall, γCD promoted a more rapid lactone hydrolysis in simvastatin than in other cyclodextrins under neutral and alkaline conditions, whereas atorvastatin remained comparatively less affected.

The hydrolysis rate constant ($k_{f}$) generally decreases as the binding constant increases, as can be seen in Table 7. For example, simvastatin–RMβCD complexes exhibit both the highest $K_{1:1}$ and the lowest $k_{f}$ under physiologically relevant conditions. However, this relationship is not solely determined by the magnitude of $K_{1:1}$; the specific orientation of the drug within the cyclodextrin cavity can also influence the hydrolysis susceptibility. Thus, two complexes with similar binding affinities may have different $k_{f}\;$ values if the lactone ring is more or less exposed.

pH further modulates this chemistry. Under acidic conditions, the lactone ring becomes intrinsically less prone to hydrolysis, diminishing the relative impact of the cyclodextrin binding, even when $K_{1:1}$ is comparatively low. Conversely, at pH values near neutrality, where the lactone hydrolysis is more rapid, the cyclodextrin type and the strength of complexation seem to be more influential in controlling $k_{f}$; in particular, derivatives with enhanced hydrophobicity or favorable charge properties (e.g., RMβCD) tend to yield higher $K_{1:1}$ values, thereby offering superior stabilization. Ultimately, optimizing both the cyclodextrin type and the solution pH can maximize the lactone protection through stronger complex formation and reduced hydrolysis rates.

The cyclodextrin complexation cannot be universally relied upon to improve the chemical stability of labile drugs in aqueous media, particularly under physiologically relevant conditions. In our study, we demonstrated that while cyclodextrin (CD) complexation can enhance the stability of statin lactones under certain conditions, the overall effect is influenced by the type of CD derivative, the pH, and possibly the orientation of the drug within the CD cavity. This observation is consistent with previous findings on β-lactam antibiotics, where the hydrolysis rate of benzylpenicillin was significantly reduced under acidic conditions upon complexation with HPβCD and RMβCD; however, under neutral to alkaline conditions, HPβCD was found to accelerate hydrolysis, likely due to changes in drug ionization and the reduced affinity for the cyclodextrin cavity. Consistent with our findings, RMβCD exhibited a reduced catalytic effect under neutral to alkaline conditions [48]. This likely stems from the methylation of the OH groups, which diminishes the hydrogen-bond formation and thereby lowers the catalytic activity.

### 3.3. Phase Solubility Studies

#### 3.3.1. Aqueous Solubility of Simvastatin (Lactone) and Atorvastatin (Acid)

As shown in Table 8, the measured simvastatin solubility was roughly in agreement with that reported in previous studies. In this study, we differentiated between the lactone and acid forms. The drug solubility in water was 14.25 ± 3.46 µg/mL for the lactone form and 7.94 ± 0.15 µg/mL for the acid form. In addition, when using phosphate buffer of pH approximately 7 and an equilibration time of 5 or 20 days at room temperature (23 ± 1 °C), a complete conversion to the acid form was observed, and the reported solubility corresponds to simvastatin acid only. By contrast, the intrinsic solubility of simvastatin lactone (6.78 ± 2.39 µg/mL) was measured in a citrate buffer at pH 4.82, where only one peak corresponding to the lactone form was observed after 5 days of equilibration. These observations highlight that both the pH and equilibration time can significantly affect measured solubility values, determining whether the reported value reflects the intrinsic (if the lactone remains intact) or the apparent solubility (if partial or complete hydrolysis occurs) of simvastatin.

On the other hand, the aqueous solubility of atorvastatin in the 0.10 M phosphate buffer solution at pH 7.01 (no cyclodextrin) was 326.59 ± 10.17 µg/ mL(*n* = 3). This value generally aligns with earlier findings, where it was 55.33 ± 0.66 µg/mL or 45.11 ± 0.16 µg/mL in the phosphate buffer (pH 6.80) after 4 days of equilibration [49,50]. In our study, a slightly higher pH (7.01) and a longer equilibration time of 5 days likely contributed to the increased measured solubility. Additionally, factors such as the ionic strength, temperature, and agitation speed may explain the discrepancies between our results and those reported in the literature.

**Table 8:** Comparison of simvastatin solubility across studies (*n* = 3).

Study	Quantified Form of Simvastatin	Medium	pH	Temperature, °C	Equilibration Time	Solubility, µg/mL
**[51]**	-	water	-	37 ± 0.5	After 2 days of equilibration, 20 RPM	9.22 ± 1.5
**[51]**	-	0.01 M phosphate buffer	7.0	37 ± 0.5	After 2 days of equilibration, 20 RPM	32.00 ± 1.8
**[52]**	-	water	-	25 ± 0.5	After 1 day of equilibration, 100 RPM	6.22 ± 2.39
**[53]**	-	water	-	21 ± 1	After 1 day of equilibration	1.394 ± 0.03
**[53]**	-	0.01 M PBS buffer	7.4	21 ± 1	After 1 day of equilibration	2.16 ± 0.05
**[53]**	-	0.08 M HCl solution	1.1	21 ± 1	After 1 day of equilibration	3.13 ± 0.08
**Current study**	Acid	0.1 M phosphate buffer	7.0	23 ± 1	After 20 days of equilibration, 120 RPM	11.75 ± 0.13
**Current study**	Acid	0.1 M phosphate buffer	7.0	23 ± 1	After 5 days of equilibration, 260 RPM	6.94 ± 0.25
**Current study**	lactone	0.1 M citrate buffer	4.8	23 ± 1	After 5 days of equilibration, 260 RPM	6.78 ± 2.39
**Current study**	Acid	water	7.5	23 ± 1	After 5 days of equilibration, 260 RPM	7.94 ± 0.15
	Lactone	water	7.5	23 ± 1	After 5 days of equilibration, 260 RPM	14.25 ± 3.46

#### 3.3.2. Preliminary Phase Solubility Study of Simvastatin in Phosphate Buffer (0.10 M, pH 7.01)

Various cyclodextrins have been used to study the solubility enhancement of simvastatin and atorvastatin in aqueous solutions using the phase solubility method [40].

**Figure 8:**
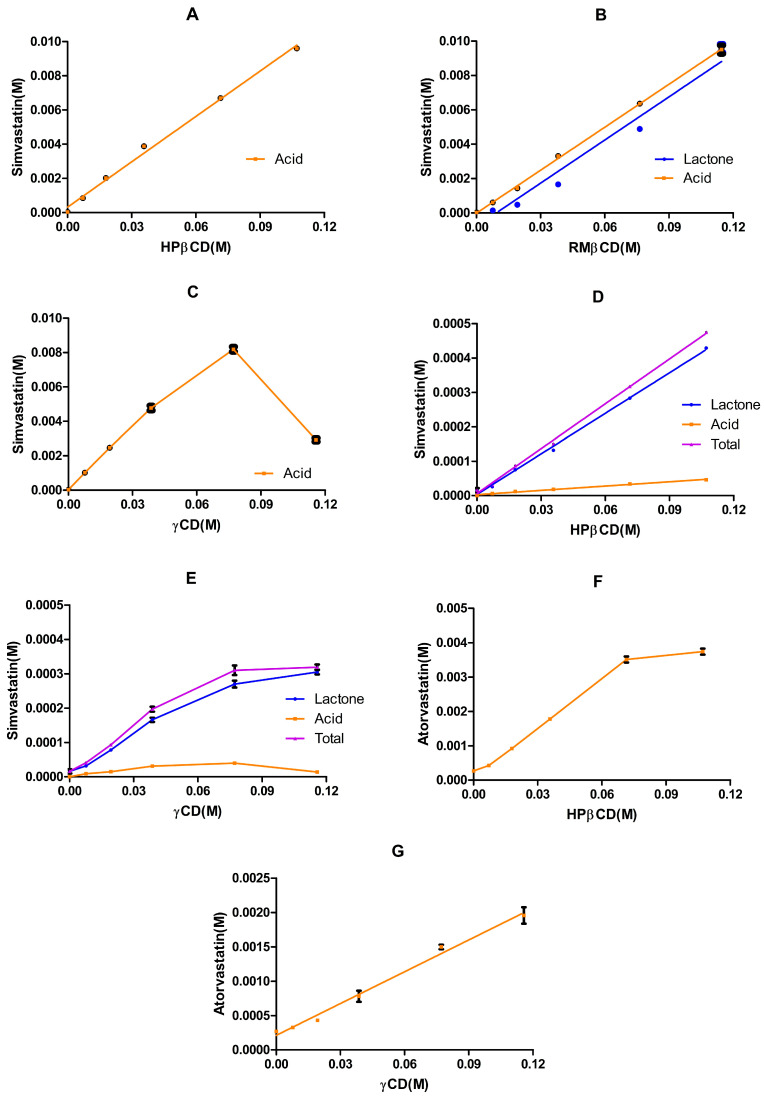
Phase solubility profiles of simvastatin and atorvastatin with various cyclodextrins and under different pH values. Panels (**A**–**C**) depict the phase profiles of simvastatin in the 0.10 M phosphate buffer (pH 7.01): (**A**) shows the profile of simvastatin (acid form) in HPβCD aqueous solutions (mean ± SD, *n* = 2); (**B**) represents the profile of simvastatin in RMβCD aqueous solutions (mean ± SD, *n* = 3); and (**C**) displays the profile of simvastatin in γCD aqueous solutions (mean ± SD, *n* = 3). Panels (**D**,**E**) depict the phase profiles of simvastatin in the 0.10 M citrate buffer (pH 4.82): (**D**) shows the solubility profile of simvastatin in HPβCD aqueous solutions (mean ± SD, *n* = 3) and (**E**) shows the overall solubility profile of simvastatin in γCD aqueous solutions over the examined concentration range (mean ± SD, *n* = 3). Panels (**F**,**G**) depict the phase solubility profiles of atorvastatin in the 0.1 M phosphate buffer (pH 7.01): (**F**) shows the overall solubility profile of atorvastatin in HPβCD aqueous solutions over the examined concentration range (mean ± SD, *n* = 3) and (**G**) shows the phase solubility of atorvastatin in γCD aqueous solutions (mean ± SD, *n* = 3).

Our preliminary phase solubility study involved equilibrating simvastatin for 20 days in buffered solutions (pH 7.01) of HPβCD (Figure 8A), RMβCD (Figure 8B), or γCD (Figure 8C). The results revealed the significantly enhanced solubility of simvastatin in the presence of these cyclodextrins. In the case of HPβCD, the phase solubility profile exhibited $A_{L}$-type characteristics: a linear increase in solubility with increasing HPβCD concentrations. Given the good linearity ($R^{2}$ = 0.9954 with a slope of 0.088), a 1:1 complexation between simvastatin and HPβCD can be assumed.

These results agreed with those of previous studies [36,53]. However, the stability constant of the complex obtained in this study was higher than that obtained in [36], and almost double the reported value in [53]. One reason for this inconsistency is that the calculation of the stability constant considered both the slope of the phase solubility profile and the apparent solubility of simvastatin, which varied among studies (Table 8). In addition, different equilibration times with varied study conditions (pH, buffer, stirring, and temperature) lead to varied rates of lactone hydrolysis and, therefore, affect the overall solubility. In our study, the complete hydrolysis of simvastatin lactone to simvastatin acid was observed. The presence of the HPβCD along with the extended equilibration time, a pH of 7.01, and a temperature of 23 ± 1 °C collectively shifted the simvastatin lactone/acid equilibrium to favor the acid form (Table 9).

Similarly, a complete conversion was observed with γCD. The phase solubility profile followed the $B_{s}$ type (Figure 8C), which is characteristic of natural cyclodextrins [54]. Additionally, based on the data in the linear part of the diagram, a 1:1 complexation between simvastatin and γCD can be assumed (good linearity: $R^{2}$ = 0.9926, with a slope of 0.1056). An even higher stability constant was obtained with γCD than with HPβCD. The parameters calculated from the slope of the initial linear portion of the SVA concentration against the γCD concentration profile assumed a 1:1 SVA–γCD complex formation.

A different scenario was obtained with RMβCD, where both simvastatin lactone and acid were detected. The phase solubility profile exhibited $A_{L}$-type characteristics for both lactone and acid forms. A 1:1 complexation between simvastatin lactone and RMβCD or simvastatin acid and RMβCD can be assumed. The stability constant of simvastatin lactone/RMβCD was bigger than that of simvastatin acid/RMβCD, and this can be explained by the smaller apparent solubility of the lactone form. Interestingly, the stability constant with RMβCD was around four times higher than that observed with HPβCD, aligning with previous findings [53], where the stability constant with methyl-β-cyclodextrin (MeβCD) was reported to be around three times higher than with HPβCD. This difference can be attributed to the increased hydrophobicity of the cyclodextrin core, resulting from the methylation of hydroxyl groups, which enhances its affinity for lipophilic drug molecules [55].

Overall, these preliminary phase solubility results for simvastatin in various cyclodextrin aqueous solutions were pivotal, as they demonstrated that simvastatin lactone is chemically unstable after 7 days or 20 days (Figure 8A–C) of equilibration at pH 7.01 and 23 ± 1 °C. Motivated by these observations, we conducted a more comprehensive chemical stability investigation employing two statin models (simvastatin and atorvastatin) with various cyclodextrin types across a range of pH levels. Based on these stability results, we subsequently conducted phase solubility studies for simvastatin under mildly acidic conditions (pH 4.82) and for atorvastatin under neutral conditions (pH 7.01).

#### 3.3.3. Phase Solubility Study of Simvastatin in Citrate Buffer (0.1 M, pH 4.82)

The phase solubility profiles of simvastatin in HPβCD and γCD aqueous solutions in a 0.1 M citrate buffer of pH 4.82 and incubated at 23 ± 1 °C for 5 days are shown in Figure 8D,E. The solubility of simvastatin under the same conditions without cyclodextrin was 6.78 ± 2.39 µg/ mL (Table 8). Overall, two different types of phase solubility profiles were obtained: $A_{L}$ was observed with HPβCD and $B_{s}$ was observed with γCD. The $R^{2}$ obtained with HPβCD was greater than 0.99 and was equal to 0.99 with the linear part of γCD. Thus, both are considered $A_{L}$ and the calculation of $K_{1:1}$ is based on Equation (1), where $S_{0}$ represents the apparent intrinsic solubility of the drug when no cyclodextrin is present in the medium [20]. The estimated parameters in Table 10 ($K_{1:1}\;and\;CE$) were determined using the slope of the linear graph (whole data) of simvastatin vs. HPβCD and the slope of the linear part of the simvastatin vs. γCD graph.

(1) $$K_{1:1}=\frac{slope\;}{S_{0}\left(1-slope\right)}$$

(2) $$CE=\frac{slope\;}{\left(1-slope\right)}$$

In general, the results obtained with HPβCD were in agreement with previous reports [34,36,53], where the reported stability constant was 571 $M^{-1}$, 774$M^{-1}$, or 1403 $M^{-1}$, whereas the value obtained in our study was 270 $M^{-1}$. However, for a poorly soluble drug like simvastatin, the observed $K_{1:1}$ might not accurately represent the true stability constant for the formed inclusion complex, as its calculation depends on the $S_{0}$, which can be influenced by various factors (pH, temperature, etc.) [56]. For example, the observed $K_{1:1}$ in our preliminary phase solubility study was 3000 $M^{-1}$ (Table 9). In contrast, the complexation efficiency (CE) appears to be more representative, as it remains independent of both the apparent and intrinsic solubility [56]. However, HPβCD appears to have a higher stability constant $K_{1:1}$ and complexation efficiency (CE) compared with γCD.

#### 3.3.4. Phase Solubility of Atorvastatin in 0.1 M Phosphate Buffer (pH 7.0)

The phase solubility profiles of atorvastatin in HPβCD and γCD aqueous solutions in the 0.1 M phosphate buffer (pH 7.01) and incubated at 23 ± 1 °C for 5 days are shown in Figure 8F,G. The solubility of atorvastatin under the same conditions (no cyclodextrin in the medium) was 326.59 ± 10.17 µg/mL. Two different types of phase solubility profiles were obtained: $A_{L}$ was observed with γCD and $A_{N}$ was observed with HPβCD. The $R^{2}$ obtained with the linear parts of HPβCD and γCD was bigger than 0.99, indicating an $A_{L}$ type; thus, the stability constant and the complexation efficiency can be determined using Equations (1) and (2), assuming a 1:1 complexation with atorvastatin. As seen with simvastatin, HPβCD appears to have a higher stability constant $K_{1:1}$ and complexation efficiency (CE) compared with γCD. The observed stability constant with HPβCD was previously reported to be 102 $M^{-1}$ [57] or 15.3 ${\;M}^{-1}\;$ [58], which is in agreement with our results (Table 11). The differences in the reported values could be associated with the apparent or intrinsic solubility of the drug. Moreover, the overall phase solubility profile with HPβCD was considered $A_{N}$ [58], where the cyclodextrin is less effective at higher concentrations, which agrees with the phase solubility diagram obtained in our study (Figure 8F).

## 4. Discussion

### 4.1. Influence of pH on Lactone–Acid Equilibrium

Under strong acidic conditions, simvastatin and atorvastatin rapidly interconvert between their lactone and acid forms, reaching an equilibrium within a few hours, with the acid form being favored at approximately 60–65%, regardless of the statin’s initial form (Figure 2I). In contrast, under neutral or alkaline pH conditions, this equilibrium is significantly diminished, and the conversion from lactone to acid appears to be irreversible, with acid being the predominant form (Figure 2III,IV). Under mildly acidic conditions (pH 4.5), lactone degradation is substantially slower, delaying the formation of the acidic form (Figure 2II). This highlights the critical role of pH in controlling the statin stability and may inform formulation strategies aimed at optimizing therapeutic efficacy.

### 4.2. Influence of Cyclodextrins on Statin Hydrolysis

Different types of cyclodextrins have varying effects on the conversion of lactone to acid; in particular, γCD and HPβCD tend to act as hydrolysis catalysts for both simvastatin and atorvastatin, especially under alkaline conditions. This catalytic effect was remarkably pronounced for atorvastatin lactone at pH 7 and above (Figure 4III,IV). However, RMβCD seemed to exert a more pronounced stabilizing effect on the lactone form in both statins (Figure 5(IIA−IVA)). This can be explained by the methylation of OH groups and their hydrophobic core. Together, these findings reveal how carefully tuned cyclodextrin selection and pH conditions can direct the pace and extent of the statin interconversion, informing formulation strategies for enhanced stability and efficacy in ocular or other targeted drug delivery systems.

### 4.3. Influence of Cyclodextrin Concentration

Overall, increasing the cyclodextrin concentration from 5% to 10% *w*/*v* had a minimal effect on stabilizing the lactone in both simvastatin and atorvastatin. For instance, increasing the HPβCD concentration from 5% to 10% at pH 4.5 and pH 7 resulted in nearly the same hydrolysis rate constants in both statins. Meanwhile, increasing RMβCD from 5% to 10% lowered the hydrolysis rate by only 15–22% at pH 2 for both statins, approximately 30% at pH 4.5 for simvastatin, and approximately 20% at pH 7 for atorvastatin (Table 4). Likewise, increasing SBEβCD from 5% to 10% reduced the hydrolysis rate by 10–33% at pH 2 for both statins and by approximately 20% at pH 7 for atorvastatin, while showing no change at pH 4.5 and pH 7 for simvastatin or at pH 4.5 for atorvastatin (Table 5). In the case of γCD, increasing the concentration to 10% reduced the simvastatin hydrolysis by approximately 14–21% at pH 2, pH 4.5, and pH 7, with no change at pH 9.5. For atorvastatin, only an approximately 4% decrease was observed at pH 2, while a 50% increase occurred at pH 4.5, and no change was observed at pH 7.

### 4.4. Formulation Implications

The acidic forms of simvastatin and atorvastatin are more stable at pH 7 and above (Figure 2(IIIB,IVB)), indicating that formulations targeting the acidic form should maintain neutral or alkaline conditions. HPβCD seems to be more suitable for formulating atorvastatin eye drops based on the cyclodextrin complexation and safety profiles. In contrast, formulating stable aqueous solutions of simvastatin–cyclodextrin inclusion complexes is unlikely, especially at a neutral pH. However, one strategy might be to use RMβCD along with a mildly acidic pH (around pH 4.5) to yield a mixture of lactone and acid forms. This can facilitate a controlled equilibrium between the lactone and acid forms, potentially offering unique therapeutic advantages if both forms are desired. This dual effect might be achieved because RMβCD forms inclusion complexes with both the lactone and acid forms of simvastatin (Figure 8B), while the mildly acidic pH maintains the equilibrium between the two forms. Indeed, delivering both the lactone (prodrug) and acid (active) forms of simvastatin is a strategy that is already employed in intranasal delivery and brain targeting [59].

An alternative strategy might be to use HPβCD or γCD to foster a more stable complexation of the simvastatin acid form, even when starting from the lactone form. This approach is particularly attractive for conjunctival or scleral drug delivery. The enhanced hydrophilicity of the resulting complexes may improve the tissue penetration and drug availability. Combining insights from the pH effects and cyclodextrin influence can guide the design of more stable and effective statin-based formulations for ocular or other delivery routes. Notably, the present study was designed as an exploratory pre-formulation study; therefore, no formal inferential statistics were applied to compare rate differences across pH levels or cyclodextrin types. Accordingly, the kinetic trends reported here should be viewed as hypothesis-generating guidance for formulation design rather than statistically validated evidence. A follow-up study with an adequate sample size and predefined statistical endpoints is planned to confirm these preliminary observations. Furthermore, future formulation studies using CDs to form complexes with statins in the lactone form should focus on the full characterization of the complexes formed to elucidate whether the lactone ring is entirely or partially excluded from the cavity. Combined data from experimental (NMR, FTIR, DSC/TGA) and computational (DFT) approaches will inform the correlation between the CD cavity size and the substitutions of different CD derivatives (e.g., hydroxypropyl, methyl, sulfobutylether) with the specific orientation of the lactone and the ester moiety.

### 4.5. Ocular Safety and Translational Considerations

Due to their high hydrophilicity and large molecular size, CDs cannot penetrate the intact corneal or conjunctival epithelium; however, they can enhance ocular drug delivery by increasing drug availability at the ocular surface [60]. In general, CDs are considered safe excipients; however, high concentrations may lead to toxicity [61]. This risk is also dependent on the specific type of cyclodextrin used. For instance, 12.5% HPβCD was well tolerated in rabbits, whereas 5% and 12.5% DMβCD were found to be toxic to the corneal epithelium [62]. Additionally, 10% SBEβCD is reported to be non-irritant in rabbits, while 4% αCD and 5% RMβCD can cause ocular toxicity [63]. Human corneal epithelium studies showed that HPβCD, γCD, and SBEβCD were relatively safe over short contact times, while DMβCD and αCD exhibited toxic effects rapidly [64]. The observed toxicity probably stems from CDs, especially at high concentrations, forming inclusion complexes with membrane phospholipids and cholesterol, thereby compromising cell membrane integrity [65].

Although RMβCD had the most stabilizing effect, especially for simvastatin, its concentration in future formulations should be carefully selected, or it should be combined with other CDs to mitigate the irritation potential. The selection of well-tolerated CDs and the exploration of safe concentration ranges are therefore indispensable for ophthalmic formulations, and, ultimately, support the regulatory approval of CD-based statin eye drops.

## Figures and Tables

**Table 1:** Calculations of the rate constant (*k*) of the forward reaction (*k_f_*) and reverse reaction (*k_r_*) for simvastatin and atorvastatin in the 0% CD-buffered aqueous solution at pH 2, 4.5, 7, and 9.5, starting with ring-closed (lactone) or ring-opened (acid) forms.

		Simvastatin				Atorvastatin			
Drug Form	Parameter (h^−1^)	pH 2	pH 4.5	pH 7	pH 9.5	pH 2	pH 4.5	pH 7	pH 9.5
**Ring-closed**	$k_{f,lactone\;loss}$	0.100 ± 0.005	0.003 ± 0.002	0.014 ± 0.0003	0.013 ± 0.0002	0.139	0.003	0.014	-
**Ring-closed**	$k_{f,Acid\;formation}$	0.063 ± 0.004	0.025 ± 0.021	0.027 ± 0.004	0.025 ± 0.002	0.149	0.030	0.017	-
**Ring-opened**	$k_{r,Acid\;loss}$	0.056 ± 0.002	0.002 ± 0.0003	-	-	0.119	0.002	-	-
**Ring-opened**	$k_{r,Lactone\;formation}$	0.094 ± 0.006	0.033 ± 0.002	-	-	0.133	0.004	-	-

**Table 2:** Calculations of the rate constant (*k*) of the forward reaction (*k_f_*) for simvastatin and atorvastatin in 0% CD or HPβCD aqueous solutions at pH 2, 4.5, 7, and 9.5, starting with the ring-closed form (lactone).

		Simvastatin				Atorvastatin			
With/Without CD	Parameter (h^−1^)	pH 2	pH 4.5	pH 7	pH 9.5	pH 2	pH 4.5	pH 7	pH 9.5
**0% CD**	$k_{f}$	0.100 ± 0.005	0.003 ± 0.002	0.014 ± 0.0003	0.013 ± 0.0002	0.139	0.003	0.014	-
**5% HPβCD**	$k_{f}$	0.029	0.0008	0.027	0.026	0.122	0.001	0.011	-
**10% HPβCD**	$k_{f}$	0.045	0.0008	0.027	0.024	0.109	0.001	0.011	-

**Table 3:** Calculations of the forward rate constant (*k*) of the same one-way hydrolysis (lactone to acid) for simvastatin and atorvastatin at pH 2, 4.5, 7, and 9.5, starting with the ring-closed form. The $k_{f,lactone}$ was calculated from the lactone loss, while $k_{f,acid}$ was calculated from acid formation.

		Simvastatin				Atorvastatin			
Cyclodextrin % *w*/*v*	Parameter (h^−1^)	pH 2	pH 4.5	pH 7	pH 9.5	pH 2	pH 4.5	pH 7	pH 9.5
**5% HPβCD**	$k_{f,lactone}$	0.029	0.0008	0.027	0.026	0.122	0.001	0.011	-
	$k_{f,acid}$	-	0.001	0.028	0.028	0.129	0.03	0.013	-
**10% HPβCD**	$k_{f,lactone}$	0.045	0.0008	0.027	0.024	0.109	0.001	0.011	-
	$k_{f,acid}$	-	0.001	0.026	0.024	0.117	0.02	0.012	-
**5% RMβCD**	$k_{f,lactone}$	0.037	0.0007	0.001	0.002	0.097	0.0008	0.005	-
	$k_{f,acid}$	-	0.002	0.001	0.0009	0.101	0.02	0.003	-
**10% RMβCD**	$k_{f,lactone}$	0.029	0.0005	0.001	0.001	0.083	0.002	0.004	-
	$k_{f,acid}$	-	0.0003	0.0009	0.0006	0.083	0.02	0.002	-
**5% γCD**	$k_{f,lactone}$	0.059	0.001	0.044	0.037	0.121	0.006	0.014	-
	$k_{f,acid}$	-	0.001	0.042	0.040	0.153	0.007	0.013	-
**10% γCD**	$k_{f,lactone}$	0.051	0.0008	0.035	0.037	0.116	0.009	0.014	-
	$k_{f,acid}$	-	0.0009	0.038	0.033	0.130	0.006	0.011	-
**5% SBEβCD**	$k_{f,lactone}$	0.030	0.002	0.009	0.009	0.365	0.002	0.005	-
	$k_{f,acid}$	-	0.002	0.009	0.009	0.455	0.002	0.004	-
**10% SBEβCD**	$k_{f,lactone}$	0.024	0.002	0.009	0.007	0.328	0.002	0.004	-
	$k_{f,acid}$	-	0.003	0.009	0.008	0.382	0.002	0.004	-

**Table 4:** Calculations of the rate constant (*k*) of the forward reaction (*k_f_*) for simvastatin and atorvastatin in 0% CD or RMβCD aqueous solutions at pH 2, 4.5, 7, and 9.5, starting with the ring-closed form (lactone).

		Simvastatin				Atorvastatin			
With/Without CD	Parameter (h^−1^)	pH 2	pH 4.5	pH 7	pH 9.5	pH 2	pH 4.5	pH 7	pH 9.5
**0% CD**	$k_{f}$	0.100 ± 0.005	0.003 ± 0.002	0.014 ± 0.0003	0.013 ± 0.0002	0.139	0.003	0.014	-
**5% RMβCD**	$k_{f}$	0.037	0.0007	0.001	0.002	0.097	0.0008	0.005	-
**10% RMβCD**	$k_{f}$	0.029	0.0005	0.001	0.001	0.083	0.002	0.004	-

**Table 5:** Calculations of the rate constant (*k*) of the forward reaction (*k_f_*) for simvastatin and atorvastatin in 0% CD or SBEβCD aqueous solutions at pH 2, 4.5, 7, and 9.5, starting with the ring-closed form (lactone).

		Simvastatin				Atorvastatin			
With/Without CD	Parameter (h^−1^)	pH 2	pH 4.5	pH 7	pH 9.5	pH 2	pH 4.5	pH 7	pH 9.5
**0% CD**	$k_{f}$	0.100 ± 0.005	0.003 ± 0.002	0.014 ± 0.0003	0.013 ± 0.0002	0.139	0.003	0.014	-
**5% SBEβCD**	$k_{f}$	0.03	0.002	0.009	0.009	0.365	0.002	0.005	-
**10% SBEβCD**	$k_{f}$	0.02	0.002	0.009	0.007	0.328	0.002	0.004	-

**Table 6:** Calculations of the rate constant (*k*) of the forward reaction (*k_f_*) for simvastatin and atorvastatin in 0% CD or γCD aqueous solutions at pH 2, 4.5, 7, and 9.5, starting with the ring-closed form (lactone).

		Simvastatin				Atorvastatin			
With/Without CD	Parameter (h^−1^)	pH 2	pH 4.5	pH 7	pH 9.5	pH 2	pH 4.5	pH 7	pH 9.5
**0% CD**	$k_{f}$	0.100 ± 0.005	0.003 ± 0.002	0.014 ± 0.0003	0.013 ± 0.0002	0.139	0.003	0.014	-
**5% γCD**	$k_{f}$	0.059	0.001	0.044	0.037	0.121	0.006	0.014	-
**10% γCD**	$k_{f}$	0.051	0.0008	0.035	0.037	0.116	0.009	0.014	-

**Table 7:** Complexation constants $K_{1:1}$ and hydrolysis rate constants $k_{f}$ for statin–cyclodextrin complexes at different pH values. $k_{f}$ values correspond to 5% (*w*/*v*) cyclodextrin.

Drug–CD Complex	$K_{1:1}$$\;(M^{-1}$)	$k_{f}$ (h^−1^)	pH (Phase Solubility Study)	pH (Chemical Stability Study)
**Simvastatin–HPβCD**	3000	0.027	7.0	7.0
**Simvastatin–γCD**	3500	0.044	7.0	7.0
**Simvastatin–RMβCD**	11,300	0.001	7.0	7.0
**Simvastatin–HPβCD**	270	0.0008	4.8	4.5
**Simvastatin–γCD**	244	0.001	4.8	4.5
**Atorvastatin–HPβCD**	164	0.011	7.0	7.0
**Atorvastatin–γCD**	56	0.014	7.0	7.0

**Table 9:** Estimated phase solubility parameters of simvastatin–cyclodextrin structures in 0.1 M phosphate buffer at pH 7.01 after 20 days of equilibration.

Simvastatin–Cyclodextrin	pH	$S_{0}$ (M)	PS-Type	$K_{1:1}$ (M^−1^)	CE
**SVA–HPβCD**	7.05 ± 0.2	0.000027	$A_{L}$	3000	0.097
**SVA–RMβCD**	6.82 ± 0.18	0.000027	$A_{L}$	2800	0.091
**SVT–RMβCD**	6.82 ± 0.18	0.000009	$A_{L}$	8500	0.091
**SVA–γCD**	6.85 ± 0.12	0.000027	$B_{s}$	3500	0.118

**Table 10:** Estimated phase solubility parameters of simvastatin in 0.1 M citrate buffer (pH 4.8).

Formulation	pH	$S_{0}$ (M)	PS-Type	$K_{1:1}$ (M^−1^)	CE
**SVT–HPβCD**	4.8	0.000016	$A_{L}$	244	0.0039
**(SVT+SVA)–HPβCD**	4.8	0.000016	$A_{L}$	270	0.0044
**SVT–γCD**	4.8	0.000016	$B_{s}$	212	0.0034
**(SVT+SVA)–γCD**	4.8	0.000016	$B_{s}$	244	0.0039

**Table 11:** Estimated phase solubility parameters of atorvastatin in 0.1 M phosphate buffer (pH 7.0, *n* = 3).

Formulation	pH	$S_{0}$ (M)	PS-Type	$K_{1:1}$ (M^−1^)	CE
**Atorvastatin–HPβCD**	6.9	0.000270	$A_{N}$	164	0.048
**Atorvastatin–γCD**	6.9	0.000270	$A_{L}$	56.3	0.016

## Data Availability

Data will be made available upon request.

## Abbreviations

The following abbreviations are used in this manuscript:

CD	Cyclodextrin
RMβCD	Randomly methylated β-cyclodextrin
HPβCD	2-hydroxypropyl-β-cyclodextrin
γ-CD	γ-cyclodextrin
SBEβCD	Sulfobutylether β-cyclodextrin
MeβCD	Methyl-β-cyclodextrin
AMD	Age-related macular degeneration
RPE	Retinal pigment epithelium
BCS	Biopharmaceutics Classification System
SVT	Simvastatin
SVA	Simvastatin hydroxyacid
HPLC	High-performance liquid chromatography
DFT	Density functional theory
CE	Complexation efficiency
